# Neurocognitive Outcome of Children Exposed to Perinatal Mother-to-Child Chikungunya Virus Infection: The CHIMERE Cohort Study on Reunion Island

**DOI:** 10.1371/journal.pntd.0002996

**Published:** 2014-07-17

**Authors:** Patrick Gérardin, Sylvain Sampériz, Duksha Ramful, Brahim Boumahni, Marc Bintner, Jean-Luc Alessandri, Magali Carbonnier, Isabelle Tiran-Rajaoefera, Gilles Beullier, Irénée Boya, Tahir Noormahomed, Jocelyn Okoï, Olivier Rollot, Liliane Cotte, Marie-Christine Jaffar-Bandjee, Alain Michault, François Favier, Monique Kaminski, Alain Fourmaintraux, Xavier Fritel

**Affiliations:** 1 CHU de La Réunion Saint-Denis/Saint-Pierre, La Réunion, France; 2 INSERM CIC-EC (CIE2), Saint-Pierre, La Réunion, France; 3 INSERM UMRS 953, “Epidemiological Research Unit on Perinatal Health and Women and Children Health”, UPMC Université Paris 6, Paris, France; 4 GRI, Research Group on Immunopathology and Infection, EA4517, Université de La Réunion, INSERM UMRS 945 “Immunity and Infection” Saint-Denis, La Réunion, France; 5 Centre Hospitalier Gabriel Martin, Saint-Paul, La Réunion, France; 6 Centre Hospitalier de l'Est Réunion, Saint-Benoît, La Réunion, France; 7 Clinique Sainte-Clotilde, Sainte-Clotilde, La Réunion, France; 8 Clinique Durieux, Le Tampon, La Réunion, France; 9 Centre d'Action Médico-Sociale Précoce (CAMSP), Saint-Louis, La Réunion, France; 10 Poitiers University Hospital, Poitiers, France; 11 INSERM CIC-P 0802, Poitiers, France; Centers for Disease Control and Prevention, United States of America

## Abstract

**Background:**

Little is known about the neurocognitive outcome in children exposed to perinatal mother-to-child Chikungunya virus (p-CHIKV) infection.

**Methods:**

The CHIMERE ambispective cohort study compared the neurocognitive function of 33 p-CHIKV-infected children (all but one enrolled retrospectively) at around two years of age with 135 uninfected peers (all enrolled prospectively). Psychomotor development was assessed using the revised Brunet-Lezine scale, examiners blinded to infectious status. Development quotients (DQ) with subscores covering movement/posture, coordination, language, sociability skills were calculated. Predictors of global neurodevelopmental delay (GND, DQ≤85), were investigated using multivariate Poisson regression modeling. Neuroradiologic follow-up using magnetic resonance imaging (MRI) scans was proposed for most of the children with severe forms.

**Results:**

The mean DQ score was 86.3 (95%CI: 81.0–91.5) in infected children compared to 100.2 (95%CI: 98.0–102.5) in uninfected peers (*P*<0.001). Fifty-one percent (n = 17) of infected children had a GND compared to 15% (n = 21) of uninfected children (*P*<0.001). Specific neurocognitive delays in p-CHIKV-infected children were as follows: coordination and language (57%), sociability (36%), movement/posture (27%). After adjustment for maternal social situation, small for gestational age, and head circumference, p-CHIKV infection was found associated with GND (incidence rate ratio: 2.79, 95%CI: 1.45–5.34). Further adjustments on gestational age or breastfeeding did not change the independent effect of CHIKV infection on neurocognitive outcome. The mean DQ of p-CHIKV-infected children was lower in severe encephalopathic children than in non-severe children (77.6 versus 91.2, *P*<0.001). Of the 12 cases of CHIKV neonatal encephalopathy, five developed a microcephaly (head circumference <−2 standard deviations) and four matched the definition of cerebral palsy. MRI scans showed severe restrictions of white matter areas, predominant in the frontal lobes in these children.

**Conclusions:**

The neurocognitive outcome of children exposed to perinatal mother-to-child CHIKV infection is poor. Severe CHIKV neonatal encephalopathy is associated with an even poorer outcome.

## Author Summary

Chikungunya virus (CHIKV), an alphaviral infection transmitted by day-biting *Aedes* mosquitoes, is widespread in Asia and in Africa. Usually, CHIKV causes a self-limiting arthritide, except in debilitated people and in neonates, for whom it can lead to severe disease. Mother-to-child perinatal transmission of CHIKV is a rare event that can occur in the setting of large-scale outbreaks when the risk of maternal viremia at the term of pregnancy becomes non-negligible. In that event, CHIKV can give rise to neonatal infection with a probability of 50%, and prostration and encephalopathy, the two leading clinical pictures in the neonate, represent a continuum in the gradation of an undiscovered central nervous system involvement. We have followed-up 33 children infected at birth between June 2005 and April 2006 on the island of La Réunion, Indian Ocean, and 135 uninfected controls, for assessing their neurodevelopmental performance around the age of two years. Fifty-one percent of infected children had a global neurodevelopmental delay compared to 15% of uninfected peers. Multivariate analysis and neuroradiology suggest without irrefutable evidence a causal relationship between CHIKV infection and neurocognitive outcomes. Our findings suggest that CHIKV infection, acquired in the perinatal period, can cause severe disease with lifelong expected disability.

## Introduction

Chikungunya virus (CHIKV), a re-emerging alphavirus transmitted by *Aedes* mosquitoes, has been responsible for major epidemics in Eastern Africa, numerous islands in the Indian Ocean, India and Sri Lanka between 2004 and 2007, before spreading for the first time into the Italian province of Emilia-Romagna [1], where it provoked, in July 2007, a three-month outbreak of 257 cases [2].

In Reunion island, a French southern-hemisphere overseas department of 787,836 inhabitants, the first cases were reported in March 2005. By October 2006, the disease burden represented a seroprevalence rate of 38.2% (300,000 infections) [3].

This outbreak drastically changed the paradigm of Chikungunya, previously seen as a non-fatal benign illness, highlighting atypical and severe forms with case fatalities, as well as long-term community burden in rheumatology, rehabilitation neurology, and sensorineural health [4]–[7].

Among these severe forms, perinatal mother-to-child CHIKV infections [8]–[12], gained the attention of caregivers and public health stakeholders, highlighting the severity of this emerging infectious disease, henceforth perceived as a global concern with the potential for lifelong consequences [6].

The spectrum of neonatal CHIKV infection included central nervous system (CNS) anomalies, hemorrhagic and cardiac manifestations [8]–[11]. CHIKV encephalopathy was associated with brain swelling (early cytotoxic and late vasogenic cerebral edema) and the presence of viral genoma in the cerebrospinal fluid (CSF), also found in CHIKV non-encephalopathic form (*i.e*, neonatal prostration). These data raised questions about the primary nature of the neurologic manifestations accompanying CHIKV infection (neurovirulence or CHIKV-specific neonatal encephalitis, or non-specific encephalopathy?) [8]–[9]. Conversely, neonates born from pregnant women who exhibited signs of CHIKV infection long before delivery, were apparently healthy at birth, and intrauterine infection was considered exceptional [9]–[12].

Prior to this study, the long-term consequences for children exposed *ante* or *peripartum* to this potential neurotropic virus were unknown. The purpose of this multi-centre ambispective cohort study was to determine whether the neurocognitive outcome of infected neonates after two years was different from that of uninfected children.

## Methods

### Population and setting

The study population consisted of neonates enrolled prospectively in the CHIMERE cohort study [12] and a cohort of previously infected neonates [8], [9]. Neonates were recruited in two different ways: 1) pregnant women attending the island's six main maternity wards between April 15, and November 2006 (78% of the 14,066 live-births in Reunion island in 2006), were invited to take part in the study; 2) Further cases of neonatal CHIKV infection, diagnosed before April 2006, were identified retrospectively from each maternity unit. Delivery room, obstetrical and neonatal wards are well insulated and air-conditioned, and at the peak of the CHIKV outbreak, blood-derived products were imported from a CHIKV-free area, minimizing the risk of CHIKV transmission by mosquito bite or by blood contamination.

### Exposure definition

CHIKV perinatal mother-to-child infection (p-CHIKV infection) was identified for the infants of mothers infected during pregnancy with a positive reverse transcriptase polymerase chain reaction (RT-PCR) result and/or presence of CHIKV-specific MAC-ELISA IgM antibodies before day 10 (or day 15 in the CSF). These children were designated as exposed-infected (EI). Infected neonates were subsequently classified as severe or non-severe based on convulsions, coma requiring mechanical ventilation, or abnormal magnetic resonance imaging (MRI) scans indicative of brain swelling (cytotoxic or vasogenic cerebral edema) during the acute phase of the disease (table S1) [8], [9]. Neonates unable to be breastfed or bottle-fed, previously defined by pediatricians as “mild prostrated” [9], or neonates requiring ventilation support for sedation and analgesia were assigned to the non-encephalopathic group with infants showing no symptoms compatible with encephalopathy-related neurologic complications.

A major issue was how to define the status of children exposed to maternal infection and testing negative for CHIKV-specific IgM antibodies at birth. Thus, *ante partum* exposure was defined for the offspring of mothers infected during pregnancy with negative RT-PCR and IgM at birth, for whom CHIKV-specific IgG seroreversed during follow-up. These children were designated as exposed-uninfected (EU). The unexposed-uninfected (UU) group comprised the children born from seronegative mothers. Other conditions diagnosed by mandatory maternal screening including toxoplasmosis, rubella, syphilis, hepatitis B virus, cytomegalovirus or overt fetal alcohol syndrome were excluded from analysis.

### Follow-up

Infants were examined by pediatricians every six months until the age of two years. MRI scans were proposed for 14 p-CHIKV-infected children with longitudinal examinations over time. The brain MRI protocol included axial and sagittal T1-weighted (T1W) imaging before and after infusion of a gadolinium-based contrast agent, T1 fast spin echo (FSE), T2-weighted (T2W), T2W echo-gradient-echo (GRE) et echo-planner imaging (EPI), diffusion-weighted (DW), apparent diffusion coefficients (ADC) mapping and spectroscopy imaging sequences.

### Outcome measures

Neurocognitive functions of children enrolled in the CHIMERE cohort were assessed around their second birthday using the Revised Brunet-Lézine (RBL) scale [13], after examination by a pediatrician to rule out acute conditions (*e.g*., infection, immunization) likely to interfere with the test. Ophthalmologic and otorhinolaryngologic check-ups had been performed previously to determine how the RBL test should be interpreted. The RBL scale is an early childhood psychomotor development scale covering four areas of neurodevelopment: movement and posture, coordination, language, and sociability (table S2). Four subscores can be calculated for children aged two to 30 months (http://mg.liens.free.fr/Pediatrie_BLR_pdf), which yield a mean global developmental quotient (DQ) of 100 with a SD of 14 [13]. The distribution of the each of the separate subscores and the global DQ are tabulated, enabling specific patterns of developmental delay to be identified. The RBL scale has been routinely used in francophone countries, as in the EPIPAGE study [14]. In our study, the test was performed by two psychometrists blinded to exposure and took account of the actual age of each child at the time of evaluation. Children were considered to have normal development if the DQ was superior to 85. Children with neurocognitive dysfunction were divided into moderate (DQ 70–85) and severe (DQ<70) global developmental delay (GND).

### Sample size

The sample size was chosen to enable the detection of a ten-point difference in DQ score between uninfected and infected children. This could be achieved with 19 EI and 76 uninfected survivors at the end of the follow-up period. This goal was impossible to achieve subsequently due to the decline of the outbreak, so we monitored all previously EI children with approximately four non-matched controls per infected child, randomly selected among the offspring of uninfected mothers or mothers infected during pregnancy without p-CHIKV infection. Matching on place of birth, gender and gestational age (GA) was planned but abandoned, given an imbalance of inclusions between centers and absence of a link between infection and preterm birth (GA<37 weeks) [8], [9], [12].

### Data analysis

Means and DQ scores were compared using parametric or non-parametric tests, as appropriate. Proportions of moderate and severe delays were compared using the Pearson χ^2^ or Fisher tests as appropriate. Indicators of GND (DQ≤85) were investigated using Poisson regression models with robust variance option to account for the cohort study design [15], controlling all the putative confounders, including a social deprivation propensity scale fitted with the five covariates of primary social determinism linked to maternal CHIKV infection (*i.e.*, maternal origin, education, marital status, parity and body mass index) [12]. Incidence rate ratios (IRR) were given with 95% confidence intervals (95% CI). Sensitivity analyses using alternative Generalized Linear Model (GLM) log-binomial, Generalized Estimating Equation (GEE)-logistic, and Cox proportional hazard multivariate regression models were performed to strengthen the results. These models have been used extensively for assessing common dichotomous outcomes (>10%) [16], while the latter was used more recently in an original way to assess the recovery of mental function after West Nile virus infection, this being considered as time-to-event binary outcome [17].

All the interaction terms between the variables included in the models were tested.

Adjusted attributable risk percents (ARP) for p-CHIKV infection and other predictors were derived from the GEE-logistic model to assess the accountability of CHIKV in the pathogenesis of GND, as follows: ARP (%) = (π_1_−π_0_/π_1_)×100, with π_1_ the prevalence of GND among infected children and π_0_ the prevalence of GND among uninfected peers. Comparisons in two-by-two adjustments and in GEE-logistic models between crude and adjusted odd ratios (OR) of p-CHIKV infection and between the variations of their standard errors (SE) allowed to distinguish confounding and over-adjustment [18], [19]. Confounding was defined using causal diagrams (directed acyclic graphs) when the adjusted OR for CHIKV infection was altered of more than 20% of its crude value. Overadjustment was defined when adjusting on the covariate altered this adjusted OR of 10–20% with a concomitant change of the SE superior than 20%. Unnecessary adjustment was considered for intermediate factors, when adjusting on the covariate did not affect the magnitude of the point-estimate but decreased its precision (SE change of 10–20%). Finally, residual confounding (RC) was addressed by measuring the inverse of the attributable risk fraction (ARF) of the pool of covariates: RC (%) = 100%−ARF (%).

All analyses were computed in Stata (release 10, StataCorp. 2008, Texas, USA) excluding observations with missing data. Statistical significance was set at *P* = 0.05.

### Ethics, funding and STROBE statement

Written informed consent was obtained from the parent/guardian. The study was approved by the ethics committee of Tours, France (no. 2006-2007) and was reported to the French Data Protection Authority. The study respected the STROBE statement (Checklist S1).

## Results

### Population characteristics

**Figure 1:**
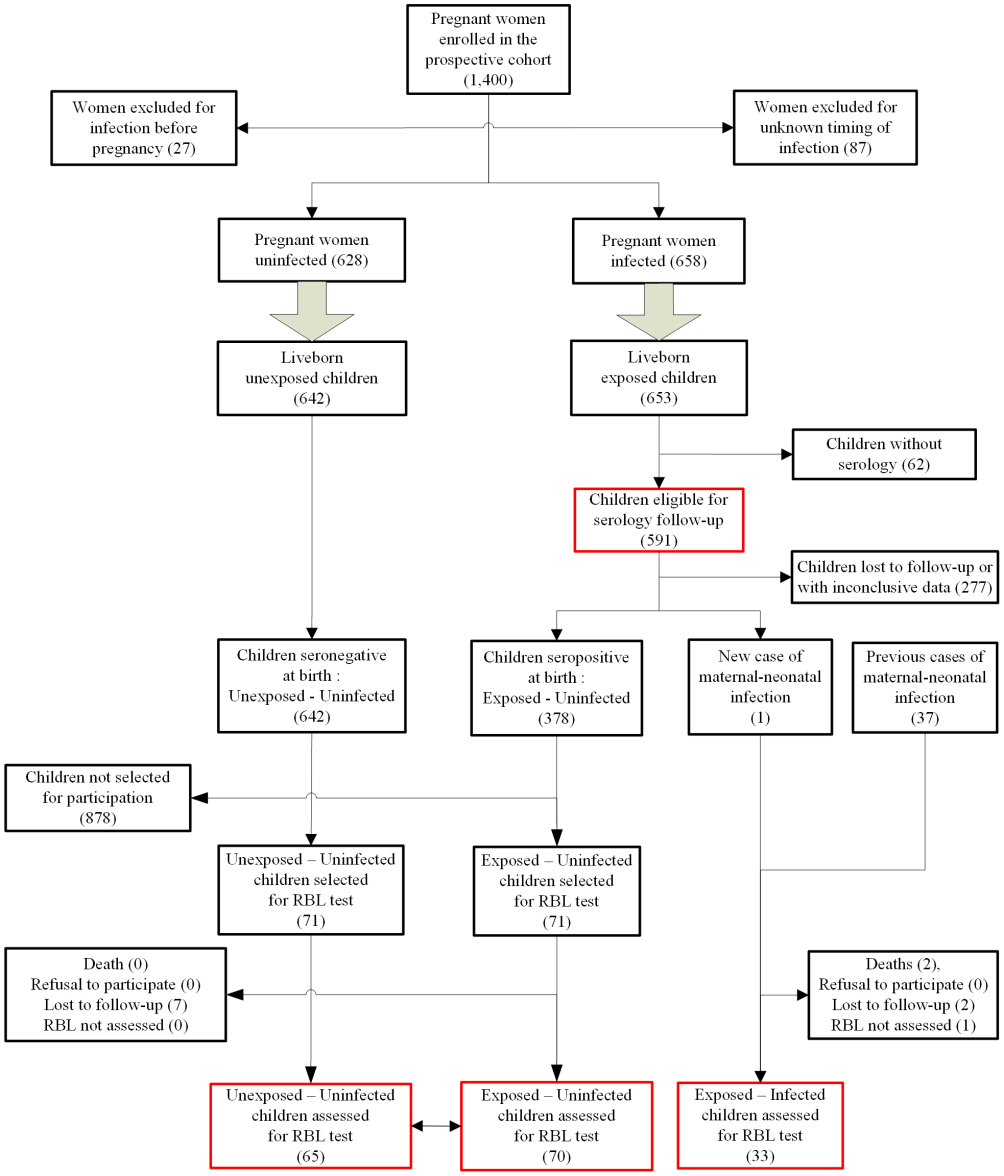
Study population. − : seronegative for CHIKV-specific IgM and IgG antibodies ; + : seropositive for CHIKV-specific IgG antibodies; M24: 24^th^ month, end of follow-up ; Unexposed - Uninfected and Exposed - Uninfected children were pooled as the Uninfected group (grey lozenge) and compared with Exposed - infected children as the Infected group (white lozenge) for RBL (Revised Brunet-Lézine) performance.

Between April 15 and August 2, 2006, we recruited 1,298 liveborn neonates in the CHIMERE cohort of whom, 653 were exposed to CHIKV during pregnancy and 591 were tested for seroreversion. The study population is presented in the figure 1.

Of the 591 children exposed to p-CHIKV infection, we observed only one case of neonatal infection *via intra partum* maternal viremia. At the same time, we enrolled the 36 survivors infected prior to April 2006. Of the 37 neonates eligible for the cohort, one family declined participation (a severe encephalopathic case who died around the age of five years in a clinical picture of cerebral palsy), two were lost to follow-up (previously described as neonatal prostration), one other could not be assessed due to severe cerebral palsy. We thus recruited 142 children (71 UU, 71 EU) to serve as non-matched controls for RBL tests, of whom seven were lost to follow-up.

The characteristics of the 168 children assessed on RBL scales are described in table 1 and in table S3. Mothers of EI children were less well educated and were more likely to live alone than mothers of EU children and far less than UU mothers [12], result shifted by a repartition bias favoring the participation of better educated mothers in the control group (differential selection of EU and UU neonates according to willingness to participate). That is why, confronted with overt social determinism of CHIKV infection [20]–[22], we then used a propensity scale of social risk of infection to control the role of maternal social characteristics on DQ while canceling repartition bias (data not shown). It should be noted that GA was lower in infected children. Shortened gestation may be due to maternal fever around full-term pregnancy, fever is known to trigger uterine contractions.

**Table 1:** Children characteristics related to perinatal mother-to-child Chikungunya virus infection, CHIMERE cohort, Reunion Island, 2008.

Exposure group	Unexposed Uninfected children (n = 65)		Exposed Uninfected children (n = 70)		Exposed Infected children (n = 33)		*P value*
Maternal characteristics							
Maternal age (μ, σ)	29.3	(±5.1)	28.7	(±6.4)	28.1	(±7.1)	0.472
Place of birth							0.168
Indian Ocean	56	(86.2)	59	(84.3)	32	(97.0)	
Mainland France	7	(10.8)	11	(15.7)	1	(3.0)	
Education							< 0.001
Primary school	14	(21.5)	34	(48.6)	19	(57.6)	
High school	19	(29.2)	21	(30.0)	10	(30.3)	
University	32	(49.2)	15	(21.4)	4	(12.1)	
Marital status							0.019
Live alone	13	(20.0)	26	(37.1)	15	(45.4)	
Lives with partner	52	(80.0)	44	(62.9)	18	(54.5)	
Parity							0.359
0	27	(41.5)	19	(27.1)	11	(33.3)	
1	20	(30.8)	31	(44.3)	13	(39.4)	
2	11	(16.9)	11	(15.7)	2	(6.0)	
≥3	7	(10.8)	9	(12.9)	6	(18.2)	
Pre-pregnancy body mass index							0.059
<25	44	(67.7)	36	(51.4)	17	(51.5)	
25–29.9	14	(21.5)	12	(17.1)	8	(24.2)	
≥30	7	(10.8)	22	(31.4)	8	(24.2)	
Smoking during pregnancy							0.150
Yes	55	(84.6)	58	(82.9)	30	(90.9)	
No	9	(13.8)	12	(17.1)	1	(3.0)	
5-itemsocial deprivation score†							< 0.001
Low (−1 to 0 point)	23	(35.4)	8	(11.4)	4	(12.1)	
Moderate (1 to 2 points)	35	(53.8)	37	(52.9)	12	(36.4)	
High (3 to 7 points)	7	(10.8)	25	(35.7)	17	(51.5)	

Neonatal characteristics	UU children (n = 65)		EU children (n = 70)		EI children (n = 33)		
Gestational age (μ, σ)	38.97	±1.99	39.51	±1.68	38.06	±1.29	0.001
Preterm birth‡							0.250
Yes	7	(10.8)	3	(4.3)	4	(12.1)	
No	58	(89.2)	67	(95.7)	29	(87.9)	
Small for gestational age#							0.650
Yes	12	(18.5)	10	(14.3)	7	(21.2)	
No	53	(81.5)	60	(85.7)	26	(78.8)	
5-minute Apgar score (μ, σ)	9.89	±0.40	9.75	±0.81	9.75	±0.61	0.525
Breastfeeding at discharge							0.003
Yes	52	(80.0)	50	(71.4)	15	(45.4)	
No	13	(20.0)	19	(27.1)	17	(51.5)	
Head growth z-score (μ, σ)	0.15	±0.96	0.09	±0.92	−0.37	±1.04	0.019
Head circumference*							0.004
−1 S.D≤z-score<+2 S.D	56	(86.2)	61	(87.1)	26	(78.8)	
−2 S.D≤z-score<−1 S.D	1	(1.5)	2	(2.9)	1	(3.0)	
z-score<−2 S.D	0	(0.0)	0	(0.0)	4	(12.1)	

Data are means, standard errors, numbers and percentages. *P* values are given for Kruskal-Wallis and Fisher exact tests comparing the three groups.

† This propensity score is derived from maternal population (see table 2 of ref. [12]) assigning positive or negative points to rounded-value beta coefficients associated with categories of maternal origin, education, marital status, parity and body mass index;

‡ gestational age <37 weeks;

# <10^th^ percentile of AUDIPOG network growth charts;

*corrected for 24 months postnatal age.

### DQ scores and mental delays following exposure

RBL tests were performed on average at 21 months of age (range 15.8 to 27 months, Kruskal-Wallis test, *P* = 0.420) and within close timeframes for the three groups of neonates (respective ranges: 18.3 to 27 months in UU children, 19.4 to 26.8 months in EU children, 15.8 to 26.7 months in EI children, Bartlett's test for equal variances, *P* = 0.011). Overall, mean DQ scores and the rate of GND (DQ≤85) in UU children were not different from those observed in EU children and within normal ranges (table 2). EU and UU children were therefore pooled together for further analysis of DQ scores and neurocognitive function. Global DQ scores were lower in infected children than among uninfected peers (EU or UU).

**Table 2:** Revised Brunet-Lezine development quotient scores related to perinatal mother-to-child Chikungunya virus infection, CHIMERE cohort, Reunion Island, 2008.

Exposure group	Unexposed Uninfected children (n = 65)		Exposed Uninfected children (n = 70)		Exposed Infected children (n = 33)		
DQ scores	mean	(95% CI)	mean	(95% CI)	mean	(95% CI)	*P* value
Global	100.1	(96.6–113.1)	100.4	(97.5–114.3)	86.3	(81.2–93.5)	<0.001
Movement/Posture	111.3	(107.1–123.4)	115.5	(112.0–128.5)	98.5	(91.0–105.3)	<0.001
Coordination	93.6	(90.3–107.0)	95.8	(92.4–117.1)	83.5	(76.0–90.9)	<0.001
Language	97.7	(93.1–110.9)	94.1	(90.6–108.5)	80.0	(74.8–87.5)	<0.001
Sociability	105.5	(101.0–118.2)	103.0	(99.0–116.8)	90.5	(84.2–97.5)	0.001

Developmental delays	n	(%)	n	(%)	n	(%)	*P* value
Global							<0.001## **
Severe‡	2	(3.1)	1	(1.4)	4	(12.1)	
Moderate†	10	(15.4)	8	(11.4)	13	(39.4)	
Absent	53	(81.5)	61	(87.1)	16	(48.5)	

DQ: development quotient. DQ scores were measured between 15.8 and 27 months of age. Developmental delay is moderate if. ^†^85≤DQ ≤70, severe if. ^‡^DQ score <70.

Data are means and 95% confidence intervals. *P* values are given for Kruskal-Wallis or Fisher exact tests comparing the three groups:

## *P* value<0.01 for Fisher exact test comparing unexposed uninfected *versus* infected children:

**P value<0.01 for Fisher exact test comparing exposed uninfected *versus* infected children.

Importantly, half the infected children had a GND. However, when considering specific skills separately, neurodevelopmental dysfunction was identified in 73.9% (23/33) of infected children, the areas most affected (DQ≤85) being coordination and language (n = 19), sociability (n = 12), movement/posture (n = 9).

### CHIKV infection clinical presentation and neurocognitive outcomes

Of those who could be assessed by the RBL test, 75.0% of the children with a history of CHIKV encephalopathy had a GND (50% moderate, 25% severe) compared with 38.1% of children who presented with a so-called “mild prostration” (table 3). Among these latter, there was one case of severe GND (DQ<70) and 33.3% with a moderate GND (DQ 70–85). Of note, non-severe (prostrated) children exhibited lower performances than uninfected peers. Thus, the most affected areas in CHIKV non-encephalopathic children were again coordination and language (n = 8).

**Table 3:** Neurocognitive outcomes related to the clinical presentation of perinatal mother-to-child Chikungunya virus infection, CHIMERE cohort, Reunion Island, 2008.

Clinical presentation	Uninfected children (n = 135)		Non severe prostrated children (n = 21)		Severe encephalopathic children (n = 12)		
DQ scores	mean	(95% CI)	mean	(95% CI)	Mean	(95% CI)	*P* value
Global	100.2	(98.0–102.5)	91.2	(85.4–97.1)	77.6	(68.6–86.5)	<0.001## **
Movement/Posture	113.5	(110.7–116.1)	103.5	(95.9–111.0)	89.8	(72.3–107.4)	<0.001**
Coordination	94.8	(92.4–97.1)	89.8	(83.1–96.4)	72.5	(62.3–82.7)	<0.001##
Language	95.8	(92.9–98.7)	84.1	(77.4–90.7)	72.8	(64.0–81.7)	<0.001## **
Sociability	104.2	(101.2–107.2)	94.9	(86.8–102.8)	83.0	(72.0–94.0)	<0.001# *

Developmental delays	n	(%)	n	(%)	N	(%)	*P* value
Global							<0.001*
Severe‡	1	(0.7)	1	(4.8)	3	(25.0)	
Moderate†	20	(14.8)	7	(33.3)	6	(50.0)	
Absent	114	(84.4)	13	(61.9)	3	(25.0)	
Movement/Posture							<0.001##
Severe‡	1	(0.7)	0	(0.0)	5	(41.7)	
Moderate†	7	(5.2)	4	(19.1)	0	(0.0)	
Absent	127	(94.1)	17	(80.9)	7	(58.3)	
Coordination							<0.001#
Severe‡	3	(2.2)	1	(4.8)	5	(41.7)	
Moderate†	30	(22.2)	8	(38.1)	5	(41.7)	
Absent	102	(76.6)	12	(57.1)	2	(16.7)	
Language							<0.001
Severe‡	9	(6.7)	2	(9.5)	5	(41.7)	
Moderate†	24	(17.8)	8	(38.1)	4	(33.3)	
Absent	102	(76.5)	11	(52.4)	3	(25.0)	
Sociability							0.001
Severe‡	3	(2.2)	1	(4.8)	3	(25.0)	
Moderate†	14	(10.4)	4	(19.0)	4	(33.3)	
Absent	118	(87.4)	16	(76.2)	5	(41.7)	

DQ: development quotient. DQ scores were measured between 15.8 and 27 months of age. Developmental delay is moderate if. ^†^85≤DQ ≤70, severe if. ^‡^DQ score <70.

Data are means and 95% confidence intervals. *P* values are given for Kruskal-Wallis or Fisher exact tests comparing the three groups; Mann-Whitney or Fisher exact test comparing encephalopathic *versus* non encephalopathic children:

## *P* value<0.01;

# *P* value<0.05;

Mann-Whitney or Fisher exact test comparing non encephalopathic infected *versus* uninfected children:

**P value<0.01;

*P value<0.05.

### Predictors of mental delay in bivariate analysis

The variables associated with GND are presented in table S4. Maternal smoking or drinking alcohol, before or during pregnancy, was not associated with neurodevelopmental outcome, only highly disadvantaged mothers (propensity score >2) were more likely to have a child with a GND (crude IRR 2.86, 1.04–7.84). Infected children were more likely to develop GND (crude IRR 3.31, 95%CI 1.97–5.54), irrespective of the clinical presentation (prostration: crude IRR 2.44, 95%CI 1.24–4.81; encephalopathy: crude IRR 4.92, 95%CI 2.86–8.42). Other neonatal characteristics linked to DQ score included preterm birth (crude IRR 2.06, 95%CI 1.04–4.08), breastfeeding (crude IRR 0.44, 95%CI 0.25–0.77), and head circumference at RBL test (z-score<−2 S.D, crude IRR 5.11, 95%CI 3.65–7.13).

### Predictors of mental delay in multivariate analysis

Perinatal mother-to-child CHIKV infection was an independent predictor of GND (adjusted IRR 2.79, 95%CI 1.45–5.34) after controlling maternal social situation, head circumference, small for gestational age (SGA) neonates, this latter factor being also susceptible to account for head circumference, as encountered in misdiagnosed fetal alcohol spectrum disorders (table 4). This finding was confirmed in sensitivity analysis (table S5). Adjusting for preterm birth, and breastfeeding, both on the causal pathway from infection to GND or on maternal education level alone, in addition or in separate models, did not change the association between CHIKV infection and GND.

**Table 4:** Predictors of global neurodevelopmental delay in multivariable analysis, CHIMERE cohort, Reunion Island, 2008.

Poisson regression model	Total	Children with GND†		Adjusted IRR	(95% CI)	*P* value¶
Chikungunya virus infection						
Yes	32	16	(50.0)	2.79	(1.45–5.34)	0.002
No	119	17	(14.3)	1	-	-
Head circumference*						
−1 S.D≤z-score<+2 S.D	143	28	(19.6)	1	-	-
−2 S.D≤z-score<−1 S.D	4	1	(25.0)	0.82	(0.27–2.41)	0.718
z-score<−2 S.D	4	4	(100)	2.38	(1.41–4.01)	0.001

Developmental quotients (DQ) were measured between 15.8 and 27 months of age.

† Global neurodevelopmental delay (GND) is defined for DQ≤85.

Data are numbers, percentages, adjusted IRR (incidence rate ratio) and robust SE (robust standard error).

¶ *P* values are given for adjusted Wald tests.

The model is adjusted for the social deprivation propensity score (see table 2 of ref. [14]) assigning positive or negative points to the rounded-value beta coefficients associated with categories of maternal origin, education, marital status, parity and body mass index; small for gestational age (defined for birth-weight <10^th^ percentile of AUDIPOG growth charts);

*head circumference is corrected for 24 months of postnatal age.

### Neuroradiologic outcomes

**Figure 2:**
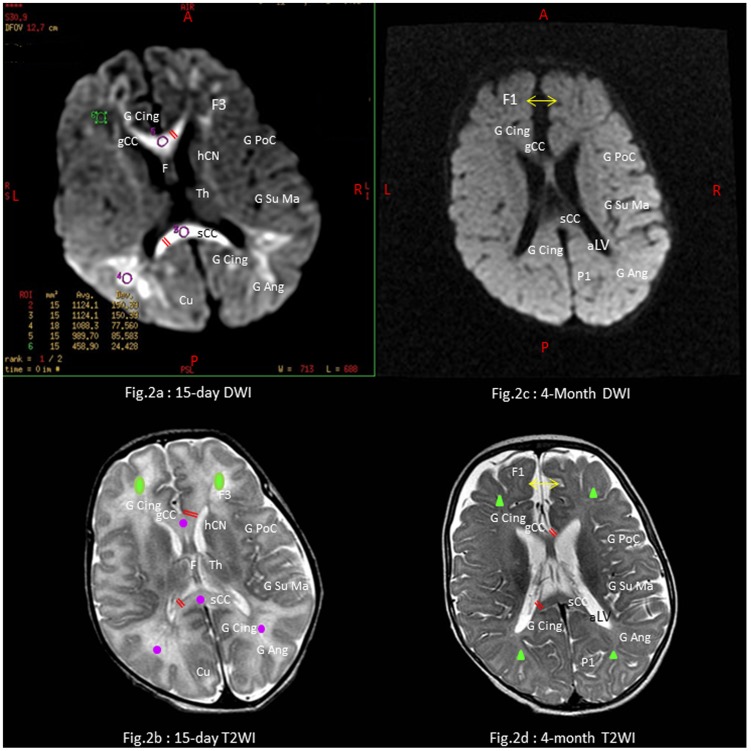
MRI scans of a four-month child with CHIKV neonatal encephalopathy. - Child n°4 of Table 5. Full-term small for gestational age 38-week neonate. m5-Apgar score: 10/10. Encephalopathy with sepsis and DIC on day 4. Global developmental delay with DQ = 77 and microcephaly (head circumference 43 cm, −1.5 z-score SD) at 20 months. Axial sections via the *interventricular foramen* at day 15 on the left side: Diffusion-weighted imaging (DWI) with apparent diffusion coefficient (ADC) map (Fig. 2a), and T2-weighted imaging (T2WI) (Fig. 2b). Axial sections via the body of third ventricular at month 4 on the right side: DWI with ADC map (Fig. 2c), and T2-weighted imaging (Fig. 2d). MRI scans show scattered areas of cytotoxic edema (violet circles) with decreased-diffusion signals on the ADC map or normal-appearing white matter (green circles) at 15-day scans, absence of persistent brain swelling (normal ADC) but scattered demyelination with scalloped-appearance of white matter atrophy (green triangles) including thinning of the *corpus callosum* (double red lines), passive dilatation of supratentorial interhemispheric subarachnoïd spaces (double yellow arrows). Anatomic abbreviations: WM: white matter; *frontal lobes:* superior frontal (F1), cingular (CingG), inferior frontal (F3), post-central (PoC), Th: thalamus, hCN: head of the caudate nucleus; *parietal lobes*: supra marginalis (SuMa), angular (Ang), superior parietal (P1) gyri; genu (gCC) and splenium (sCC) of the *corpus callosum*; occipital lobe (Cuneus); aLV: atrium of the lateral ventricule containing the choroid plexuses.

Early MRI scans taken in the two first months of CHIKV neonatal encephalopathy have been described elsewhere [8], [9], [23]. Two-week and four-month DWI and T2WI MRI scans are summarized for a full-term child in figure 2. These two phases were identified as critical in predicting neurocognitive outcome because the interval of 15 weeks between is long enough to rule out the reversible lesions of acute and subacute phases (namely, hemorrhages, cytotoxic and vasogenic edema) and to observe chronic lesions of the white matter (WM). Subsequent MRI scan features of CHIKV neonatal encephalopathy at the early chronic phase were remarkably consistent (data not shown).

Neuroradiologic features were completed to the age of two years for eight children (table 5). Though not exhaustive, this longitudinal study showed improvements in non-severe children, while persistent injury was observed in five encephalopathic children, of whom four had microcephaly.

**Table 5:** Two-year MRI scan features of CHIKV-related white matter injury in eight children with perinatal mother-to-child Chikungunya virus infection, CHIMERE cohort, Reunion Island, 2008.

Child	Initiation presentation	Age (mo)	GDQ	HC (SD)*	WM areas	Demyelination	Cavitations	Gliosis	Spectroscopy
n°1	Encephalopathy	25.9	69	−1.0	Diffuse, CC	VC	VC	OVc	Normal
n°2	Encephalopathy	24.4	84	−0.3	Diffuse	VC, OvC	Absent	Absent	Normal
n°3	Encephalopathy	20.3	74	−2.2	Diffuse, CC	VC, OvC	Absent	Absent	↓ NAA
n°4	Encephalopathy	20.2	77	−2.7	Diffuse, CC	VC, OvC	Absent	Absent	↓ NAA
n°5	Prostration	20.1	79	−0.5	Normal	Absent	Absent	Absent	Normal
n°6	Encephalopathy	20.2	NA	−3.2	Diffuse, CC	VC	VC	Absent	↓ NAA
n°7	Prostration	15.8	101	+0.1	Normal	Absent	Absent	Absent	Normal
n°8	Prostration	22.4	96	−0.1	Normal	Absent	Absent	Absent	Normal

Age at neuropsychological evaluation (months); GDQ: global development quotient; HC:

*head circumference is corrected for 24 months of postnatal age;

SD: standard deviation; WM: white matter; NA: not assessed. Diffuse includes frontal plus two or more lobes; CC: *corpus callosum* ; OC: ovale centrum ; VC: ventricular crossroads.

↓NAA : reduction of N-acetyl-aspartate peak indicative of white matter hypometabolism or axonal loss;

### Etiologic fractions of mental delay predictors

Importantly, we did not find that the effect of CHIKV infection on neurodevelopmental outcome could be confounded by all the aforementioned covariates (changes <20% between crude and adjusted OR, data not shown). Moreover, there was no significant evidence for over-adjustment (changes >20% between crude and adjusted SE, data not shown), the most unnecessary adjustment being on head circumference (table S6a), microcephaly being definitely on the causal pathway between CHIKV infection and GND (figure 2 and table 5). Surprisingly, adjusting for preterm birth did not contribute to over-adjustment (table S6b). Amongst the four covariates controlled in the two GEE-Logistic models, p-CHIKV infection exhibited the strongest etiology fractions (ARP: 65.6% in model A, or 68.2% in model B) for the independent risk of GND and taken together, these four covariates accounted for either 95% (model A) or 98% (model B) of the neurocognitive performance. Otherwise stated, nearly two thirds of the GND observed in p-CHIKV infected children enrolled in the CHIMERE cohort could be attributable to CHIKV and residual confounding, the proportion of GND not explained by the models, was estimated at 5% (model A) or at 2% (model B).

## Discussion

The CHIMERE cohort study provides the first assessment of neurocognitive functions of infants infected by maternal-fetal transmission of CHIKV at birth, on average 21 months after infection. Overall, infected children exhibit poorer neurocognitive skills than uninfected peers, as evidenced by lower global DQ scores and diminished specific neurocognitive skills, even reaching abnormal ranges for coordination and language. Thus, incidence of GND in infected children is just over 50% but with a caveat: CHIKV encephalopathy gives the poorest neurocognitive outcome and prostration also gives rise to a certain degree of neurocognitive dysfunction. Furthermore, CHIKV is an independent predictor for GND, infected children carrying a three-fold risk of GND after adjustment for maternal social situation and neonatal characteristics, such as SGA and head circumference (table 4). Foremost is the concern that CHIKV-specific neonatal prostration, which was previously thought to have a favorable outcome, is more likely to lead to GND than the absence of infection. Hence neurocognitive dysfunctions were more frequent in non-severe p-CHIKV-infected children than in uninfected peers.

Another issue of paramount importance raised by the large-scale Chikungunya epidemic in La Reunion was that protracted high fever in the mother might trigger cognitive dysfunction in the offspring. Thus, 9.8% to 18.2% of pregnant women were estimated to have been infected [3], [9]. Indeed, high fever throughout pregnancy, associated with other maternal infections, has been linked to various neurologic outcomes such as neural tube defects [24], seizures or cerebral palsy [25], [26], autism or epilepsy [27],[28], schizophrenia and Parkinson's disease [29], [30]. Maternal hyperthermia and pro-inflammatory cytokines have been consistently demonstrated in small animal models to affect apoptosis, perturbation of neuronal migration into the neocortex and down regulation of brain-specific genes involved in behavioral changes in the offspring [31], [32]. In the CHIMERE cohort, EU children presented similar DQ scores to UU children, and maternal exposure to CHIKV during pregnancy had no impact on child neurocognitive performances at around two years of age.

CHIKV has been thought to infect the CNS since the early 1960's, based on observations in South East Asia [33], but it is only since the recent large-scale epidemics in the Indian Ocean that the prevalence and pathogenesis of neurologic syndromes have been documented [23]. Neurologic involvement was first thought to be rare at population level, affecting just one per thousand infected cases in hospital emergency departments [5]. However, this figure must be reexamined in the light of the TELECHIK population-based cohort study, which found that ten percent of CHIKV patients complained of light cerebral disorders, on average eighteen months after the Reunion island outbreak [6]. Those at risk of severe neurologic damage include preterm and full-term neonates with an inefficient type-I interferon response [34], which proved relevant for the neonatal period from an epidemiologic point of view [9], [23]. Major features of severe neurologic involvement have been described mainly in the neonate [8], [9] and through infancy and childhood [35], [36], but neurologic disorders also unexpectedly emerged in healthy adults with no known immunosuppression or underlying comorbidities [4], [5]. The CHIMERE cohort provides some novel arguments in favor of CHIKV neurologic burden, showing areas of neurocognitive dysfunction for the first time. As expected from early MRI scans indicative of scattered brain swelling and WM injury [8], [9], neurocognitive dysfunction was extensive and impaired overall performance was observed in 74% of affected cases. Coordination and language skills (19/23) were most often affected while movement/posture (9/23) and sociability (12/23) appeared to be less affected. These findings are consistent with full neuroradiologic follow-up of five cases of CHIKV neonatal encephalopathy which not only showed the progression of supra-tentorial WM injury over time, but also N-acetyl-aspartate low peaks, indicating WM hypometabolism or axonal loss on spectroscopy, particularly in the frontal lobes, where coordination and language (Broca area) centers are located [23], [37]. Interestingly, movement/posture, which implies infra-tentorial networks including basal ganglia, brainstem and spinal cord structures, was less frequently impaired. The impact on sociability is difficult to interpret because this essentially prefrontal cognitive function is not mature enough at the age of the RBL test to be specifically targeted. However, poor performance in social skills observed in half the GND infected cases raises concerns for the adaptive ability of p-CHIKV-infected children and deserve further studies.

Another major concern is the development of cerebral palsy following CHIKV encephalopathy and from the negative association between p-CHIKV infection and head growth with infection impending head growth in nearly two thirds of infected children. Cases of severe disability, WM and *corpus callosum* atrophy, and widening of the ventricular crossroads, in four out five children with microcephaly following CHIKV encephalopathy reinforces the argument for causality between p-CHIKV infection, neuronal loss (due to WM damage), small head circumference (as a result of reduced brain volume), and cerebral palsy or neurocognitive dysfunction. These latter findings support the theory of neurotropism of CHIKV, without providing further irrefutable evidence, in the absence of histopathological data from child autopsy. These data are consistent with early histopathologic findings observed in an adult deceased from CHIKV encephalomyeloradiculitis whose brain was swollen and showed microglial activation and demyelination foci located in the subcortical WM [38]. This is also in line with WM injuries found in neonatal encephalitis, such as those resulting from enterovirus or parechovirus infections [39], [40]. Whether it is caused by microglia following an innate immune response possibly leading to neuronal apoptosis and axonal injury [41], [42] remains to be confirmed for CHIKV encephalopathy in humans [43]. Nevertheless, there are several arguments that this pathogenesis is shared with other viral encephalitides or periventricular leucomalacy in preterm neonates. Certainly, both conditions target the periventricular germinal areas of neuron production and repair, involve ischemic-reperfusion and inflammation mechanisms, patterns found in CHIKV encephalopathy [9], [36]. If true, the most plausible pathway for the CHIKV to disrupt the CNS would thus be to target first and foremost the richly vascularized choroïd plexuses, then the leptominges and the ependymal wall [34], [44], using monocytes to disseminate *via* the bloodstream [45]. This is supported by reduction of T1 elongation times (WM hyperintensities on T1WI scans) on MRI of a few infants (data not shown), consistent with a certain degree of microglial activation leading to demyelination [43], [46], in the vicinity of the choroïd plexuses (of which the epithelial layer lining the lateral ventricles). We hypothesize that CHIKV in targeting stromal ependymal cells could hijack stem cell production and neuron migration to damaged areas of the brain [47], and could impair myelin sheath production by down-regulating the biosynthesis or cerebral uptake of cholesterol [48]–[50], a set of conditions that would reduce WM, brain volume and lead to neurocognitive dysfunction. Indeed, demyelination, the putative hallmark of CHIKV neonatal encephalopathy might also be caused by autoreactive CD8(+)T lymphocytes to clear virally infected target cells, as observed with oligodendrocytes in several mouse models of encephalitis [51], [52], CD8(+) T cells being the most common phenotype of peripheral blood mononuclear cells found in the CSF of cynomolgus macaques, in the only nonhuman primates model challenged by CHIKV [53]. Whatever the mechanism involved in the CNS, CHIKV pathogenesis has still many gray areas, such as the precise role of brain outer cells [34], [44], microglia [42], [43], [48], the possibility of parenchymal cells invasiveness [33], [42], or primary metabolic encephalopathy [54].

Our study has some strengths and limitations. Exposure was able to identify with certainty, encephalopathy from other causes and embryofœtopathies were ruled out, unexposed children being randomly selected through the maternal CHIMERE cohort and the serological status of each infant was known [12]. The sera of exposed children were monitored until seroreversion to rule out misclassification of children infected early in pregnancy [55]. There was no residual participation bias driven by maternal situation while using a propensity scale of social disadvantage. In order to limit information bias, psychometrists were unaware of the infectious status of the children. In the absence of matching strategy, assessment of neurocognitive function took account actual age and was set-up within close timeframes to avoid temporal bias between infected and uninfected cohorts. Most of prevalent cases of neonatal infection had been diagnosed prospectively in real-time with the help of the same lab facilities [9], so that there is no recall bias affecting their classification as EI. It should be noted that the neuroradiologic monitoring was uncontrolled, incomplete, and oriented potentially towards the most severely infected children. However, the redundancy of our observations makes unlikely the spectrum of the neuropathologic findings would have not been captured. Finally, we found no imbalance in our different models between over-adjustment and residual confounding that could skew significantly the “total causal effect” of p-CHIKV infection on neurocognitive outcome. However, multivariate analysis in our study did not aim to estimate the “true force” of the infection in predicting child psychomotor impairment, but merely, to argue for causality while controlling for various accurate predictors of psychomotor development. In consequence, we can reasonably propose that CHIKV infection is the leading contributor for neurocognitive impairment in p-CHIKV-infected children and that the social disadvantage of mothers, growth retardation, or head circumference, *i.e.*, a proxy of cerebral volume, or other factors (*e.g.*, preterm birth or breastfeeding) in relation to these variables, accounted for a lesser part of this association. This being said, we advocate a caveat in the interpretation of causality: as with several arboviruses causing mild illness, but rarely encephalopathy, our data preserve a considerable room for uncertainty regarding the potential for neurovirulence of CHIKV, so that it is impossible to claim that CHIKV is responsible of poor neurodevelopmental outcome. Indeed, the possibility of neuroinvasiveness of CHIKV remains to be shown in humans.

Several lessons can be drawn from the CHIMERE cohort study. First, as for other neglected tropical diseases [20], CHIKV infection tends to impact impoverished mothers [12] and more often their children (table 1). However, mothers infected by CHIKV during pregnancy may be reassured: the prognosis of their uninfected child is identical to that of children exposed *in utero* to maternal fever, for which there is currently no convincing evidence of subsequent neurocognitive impairment. By contrast, the neurocognitive outcome of infected children is poor and must be monitored throughout childhood to anticipate the psychomotor, cognitive and behavioral therapies designed to limit the extent of neurocognitive dysfunction and the lifelong consequences of “CHIKV driven disability”. Thus, in the absence of effective postponing of delivery or caesarian section [9], these issues should prompt further research aimed at preventing or limiting infection, such as vaccination or immunotherapy. Our findings will guide clinicians, public health specialists, policy makers and other stakeholders dealing with the challenges of maternal-fetal transmission of CHIKV during large-scale outbreaks.

## Supporting Information

Table S1 **Diagnostic criteria for classifying Chikungunya virus neonatal infections, CHIMERE cohort, Reunion island, 2008.** The criteria are exclusive for discriminating the two groups. Chikungunya virus genoma in the cerebrospinal fluid could be positive in both groups and was not considered discriminant. Indicators of CSF inflammation (white blood cell ≥4/mm^3^ or protein level ≥40 mg/dL) were not considered mandatory.
(DOC)

Table S2 **English translation of the items of the Revised Brunet-Lezine scale covering the range of age-related performances on developmental quotients of the children, CHIMERE cohort, Reunion island, 2008.** A: quality of base station; V: prone; C: supine; D: standing; Q: question to parents. If no item is specified, the child is seated at the table (on the lap of a parent for infant and small children).
(DOCX)

Table S3 **Organ dysfunctions at presentation according to gestational age-specific standards related to exposure group, CHIMERE cohort, Reunion island, 2008.** ^†^ lenticulo-thalamo striatal vasculitis, frontal or parietal hyperechogenicity on head ultrasound, or scattered white matter lesions on MRI scans ^‡^ volume expansion or vasopressor amines. N.A not assessed.
(DOCX)

Table S4 **Predictors of global neurodevelopmental delay in bivariable Poisson regression analysis, CHIMERE cohort, Reunion island, 2008.** Developmental quotients (DQ) were measured between 15.8 and 27 months of age. ^§^ Global neurodevelopmental delay (GND) is defined for DQ≤85. Data are numbers, percentages, crude IRR (incidence rate ratio) and 95% confidence intervals. ^¶^ *P* values are given for crude Wald tests. ^†^ This propensity score is derived from maternal population (see table 2 of ref. [12]) assigning positive or negative points to rounded-value beta coefficients associated with categories of maternal origin, education, marital status, parity and body mass index; ^‡^ gestational age <37 weeks; ^#^<10^th^ percentile of AUDIPOG growth charts; *corrected for 24 months postnatal age.
(DOCX)

Table S5 **Predictors of global neurodevelopmental delay in three alternative multivariable regression models: GLM Log-Binomial, GEE-Logistic and Proportional hazard model, CHIMERE cohort, Reunion island, 2008.** Developmental quotients (DQ) were measured between 15.8 and 27 months of age. ^§^ Global neurodevelopmental delay (GND) is defined for DQ≤85. ^†^ see ref. [16] for precisions; ^‡^ see ref. [17] for precision. Data are numbers, percentages, adjusted RR (risk ratios), adjusted OR (odds ratios) and adjusted HR (hazard ratios), and robust SE (robust standard error). ^¶^ *P* values are given for adjusted Wald tests. The model is adjusted for the social deprivation propensity score (see table 2 of ref. [12]) assigning positive or negative points to the rounded-value beta coefficients associated with categories of maternal origin, education, marital status, parity and body mass index; small for gestational age (defined for birth-weight <10^th^ percentile of AUDIPOG growth charts); *head circumference is corrected for 24 months of postnatal age; **head growth is taken as time-to-event covariate on a clinical and statistical basis, the value of head circumference being dependent of the timing of the measure with different growth kinetics between groups.
(DOCX)

Table S6 **Etiologic fractions of the predictors of global neurodevelopmental delay and overall attributable risk fraction explained by two GEE-logistic multivariable regression models, CHIMERE cohort, Reunion island, 2008.** Developmental quotients (DQ) were measured between 15.8 and 27 months of age. ^§^ Global neurodevelopmental delay (GND) is defined for DQ≤85. Data are numbers, percentages, adjusted odd ratios (OR), standard errors (SE), attributable risk percents (ARP) and attributable risk fraction (ARF). The effect of CHIKV infection on GND is confounded for adjusted OR change >20% of its crude value (adjusted OR <4.60). The amount of residual confounding is measured as the inverse of the ARF of the model: RC = 1- ARF (%). Overmatching is defined for an adjusted OR change of 10–20% of its crude value with concomitant SE change >20% (SE out of 1.95–2.92). ^†^The models are adjusted for the social deprivation propensity score (see table 2 of ref. [12]) assigning positive or negative points to the rounded-value beta coefficients associated with categories of maternal origin, education, marital status, parity and body mass index; small for gestational age (defined for birth-weight <10^th^ percentile of AUDIPOG growth charts); *head circumference is corrected for 24 months of postnatal age or ^‡^ gestational age <37 weeks.
(DOCX)

Checklist S1 **STROBE Statement—Checklist of items that should be included in reports of** ***cohort studies.***
(PDF)
