# Supplementary material for: Neurocognitive Outcome of Children Exposed to Perinatal Mother-to-Child Chikungunya Virus Infection: The CHIMERE Cohort Study on Reunion Island
Source: PLoS Negl Trop Dis. 2014 Jul 17;8(7):e2996. doi: 10.1371/journal.pntd.0002996 (PMC4102444; doi:10.1371/journal.pntd.0002996)
Supplement: Table S1 — Diagnostic criteria for classifying Chikungunya virus neonatal infections, CHIMERE cohort, Reunion island, 2008. The criteria are exclusive for discriminating the two groups. Chikungunya virus genoma in the cerebrospinal fluid could be positive in both groups and was not considered discriminant. Indicators of CSF inflammation (white blood cell ≥4/mm3 or protein level ≥40 mg/dL) were not considered mandatory. (DOC) [file pntd.0002996.s001.doc]

Supporting file 1

| **Table S1. Diagnostic criteria for classifying Chikungunya virus neonatal infections, CHIMERE cohort, Reunion island, 2008** | | |  |
| --- | --- | --- | --- |
| **Diagnostic criteria**  **(at least one positive criterion)** | **Severe encephalopathic**  **children (n=12)** | **Non severe prostrated**  **children (n=21)** | |
| Convulsions | + | - | |
| Coma requiring mechanical ventilation | + | - | |
| Cytotoxic or vasogenic edema on early MRI scan | + | - | |
| Conscious but unable to be breastfed or bottle-fed | - | + | |
| Conscious with ventilation support for sedation and analgesia | - | + | |
