# Supplementary material for: Neurocognitive Outcome of Children Exposed to Perinatal Mother-to-Child Chikungunya Virus Infection: The CHIMERE Cohort Study on Reunion Island
Source: PLoS Negl Trop Dis. 2014 Jul 17;8(7):e2996. doi: 10.1371/journal.pntd.0002996 (PMC4102444; doi:10.1371/journal.pntd.0002996)
Supplement: Table S2 — English translation of the items of the Revised Brunet-Lezine scale covering the range of age-related performances on developmental quotients of the children, CHIMERE cohort, Reunion island, 2008. A: quality of base station; V: prone; C: supine; D: standing; Q: question to parents. If no item is specified, the child is seated at the table (on the lap of a parent for infant and small children). (DOCX) [file pntd.0002996.s002.docx]

**Supporting file 2**

| **Table S2. English translation of the items of the Revised Brunet-Lezine scale covering the range of age-related performances on developmental quotients of the children, CHIMERE cohort, Reunion island, 2008** | | | | |
| --- | --- | --- | --- | --- |
| **Age** | **Movement and posture** | **Coordination** | **Language** | **Sociability** |
| 2 months | V: Raises head and shoulders  C: retains the head right shot-sitting  C: turns prone to supine | Eyes follow a moving person  C: eyes follow the ring on 180°  Turns his head to follow an object | Responds to voice by immobilization  Vocalizes two sounds or extended vocalizations | C: smile reply  C: Shakes when you approach him |
| 3 months | A: holds her head without oscillating  V: draws on his forearms  C: raises head and shoulders shot-sitting | Stares the cube on the table  C: firmly holds the rattle placed in hand  C: plays with her hands | C: vocalizes in response to the examiner | Q: shakes when preparing the bottle  Q: laughing out loud  (Q): aware of new situations |
| 4 months | V: keeps legs extended  C: directed to put the towel on his head movements  A: holds sitting with lightweight support | Stares the pellet and follows it with eyes  C: shakes and looks at the rattle put in hand  C: leads a movement towards the ring  Grabs a cube in contact | Immediately turns his head to look at the person speaking  Q: expresses differently pleasure / displeasure, anger / shouting | Participates in physical games (laughs) |
| 5 months | C: explores his legs and knees  D: held under the arms, jumper stage | Holds a cube in his hand and looks at the second  Reaches out to the rattle kept away  Grabs a hand threw the ring in front of him | Q: laughs and vocalizes manipulating toys  Q: shows interest to outside noise | Smiles at the mirror  Q: cries when a loved one departs  Differentiate familiar and foreign faces |
| 6 months | C: gets rid of the towel  C: takes his feet in his hands  C: turns sitting  in shot-sitting | Seizes in his palm the cube on the table  Holds 2 cubes and looks at the 3rd  Grabs the tablet raking  Raises the cup by the handle back  (hidden cube) | Q: makes rolls or vocalizes four sounds  Reacts immediately to its name | Checks out what the adult looks at if this is pointed (joint attention) |
| 7 months | A: holds sitting briefly without support  A: sitting with support, removes the towel  V: rises by his knees, push his arms  C: bears his feet  in his mouth | Grabs 2 cubs,  one in each hand  Searches for the spoon fell to the ground  Looks forward to the bell | Q: attracts attention by gestures, cries or vocalizations | Actively involved in the cuckoos game  (Q): plays throw his toys |
| 8 months | V: removes the towel placed on his head  C: turns supine to prone  A: sitting without support, removes the towel | Grabs the pellet with participation of thumb  (Q): plays hitting two objects  Attracts the ring to him using the string  Rings the bell | Q: vocalizes several distinct syllables  Q: reacts to some familiar words | Manifests when putting an object out of reach |
| 9 months | V: net displacement movements  D: stands with support  D: supported under the arms, makes steps | Accepts the 3rd cube dropping of the 2  Grabs the pellet between the thumb and forefinger  Finds the toy in the towel  Removes the round of the clipboard | Q: issues redoubled syllables | Q: understands a prohibition  Q: eats with fingers or drinks alone from a maintained bottle |
| 10 months | C: Sits alone  C: standing with support, lifts one foot  D: changes from sitting to standing with support | Raises the cup and took the cube hidden below  Places a cube in the cup or withdraws it  Search the pellet through the bottle  Seeks the door of the bell | Q: speaks a two-syllable word | Looks what the adults (without being indicated)  Q: repeats its own gestures that make laugh |
| 12 months | D: control the passage from standing to sitting  without dropping  D: makes a few steps, held in a hand  D: holds standing three seconds without support | Takes the third cube without dropping  the other 2  Loose a cube in the cup  Resets the round in a hole on the clipboard  Scribbles weakly  on demonstration | Q: Shakes the head  to say "no"  Q: babbles expressively | Q: active when being dressed (holds out his hand or foot) |
| 14 months | D: walks alone fluently (five steps)  D: climbs up stairs 4-feet | Reproduces a 2-cube tower on model  Fills the cup of cubes  (at least 5)  Introduces the pellets  in the vial  Places the round in the hole on order  Makes a scribble on order | Q: uses onomatopoeia which serve to identify objects, animals, etc...  Designates 1 object among the 5 presented | Points to what interests |
| 17 months | D: walks backwards  D: shoots the ball of the foot | Reproduces a 3-cube tower  Turns the page of a book  Removes the pellet from the vial  Places the round on the clipboard turned to 180° | Q: speaks five words  Designates 3 objects among the 10 presented | Q: drinks alone with a glass held in both hands, eats alone with a spoon  Makes drink, eat or cap adults (2/3) |
| 20 months | D: gives a kick the ball away after demonstration  D: runs with coordinated movements | Reproduces a five-cube tower  Maintains the sheet with the other hand to draw  Places the 3 pieces  on the clipboard | Names 2 or designates 4 images of 6  Designates 4 objects among the 10 presented  Q: makes 2-word sentences (distorted) | sits, give to drink or brush a doll on order (2/3)  Q: pretend play  (deferred imitation) |
| 24 months | D: gives a kick in the ball on order  D: stands on one foot with the help of a hand held | Aligns the cubes to copy a 5-cube set  Mimics a line without respected direction  Places the 3 pieces  on the returned clipboard | Names 6 images of 15  Designates 8 objects or names 4 among the 10 presented  Q: makes 3-word sentences  Q: use his first name when talking from himself | Q: wash and try to wipe his hands |
| 30 months | D (Q): climbs up the stairs alone alternating feet (with or without wall support)  D: stands 2 seconds on one foot without help | Reproduces a 4-cube wall (2 over 2)  Reproduces a 8-cube tower  Mimics a horizontal and a vertical line | Names 6 images of 15  Names 8 objects among the 10 presented  Q: use pronouns "I, you, he, she" | Understands 2 prepositions among the 5 proposed (in, on, behind, in front, below)  Q: puts his slippers or socks alone |
| **NOTE.** A: quality of base station; V: prone; C: supine; D: standing; Q: question to parents. If no item is specified, the child is seated at the table (on the lap of a parent for infant and small children) | | | | |
