# Supplementary material for: Neurocognitive Outcome of Children Exposed to Perinatal Mother-to-Child Chikungunya Virus Infection: The CHIMERE Cohort Study on Reunion Island
Source: PLoS Negl Trop Dis. 2014 Jul 17;8(7):e2996. doi: 10.1371/journal.pntd.0002996 (PMC4102444; doi:10.1371/journal.pntd.0002996)
Supplement: Table S3 — Organ dysfunctions at presentation according to gestational age-specific standards related to exposure group, CHIMERE cohort, Reunion island, 2008. † lenticulo-thalamo striatal vasculitis, frontal or parietal hyperechogenicity on head ultrasound, or scattered white matter lesions on MRI scans ‡ volume expansion or vasopressor amines. N.A not assessed. (DOCX) [file pntd.0002996.s003.docx]

**Supporting file 3**

| **Table S3. Organ dysfunctions at presentation according to gestational age-specific standards related to exposure group, CHIMERE cohort, Reunion island, 2008** | | | | | | |  |
| --- | --- | --- | --- | --- | --- | --- | --- |
| **Exposure group** | **Unexposed Uninfected** | | **Exposed Uninfected** | | **Exposed Infected** | |  |
|  | **children (n=65)** | | **children (n=70)** | | **children (n=33)** | |  |
| **Organ Dysfunction** | **Yes** | **(%)** | **Yes** | **(%)** | **Yes** | **(%)** |  |
| Central nervous system (abnormal Head ultrasound or MRI scan findings) ^†^ | N.A | - | N.A | - | 12 | (36.4) |  |
| Hemocoagulation system (need for platelet or fresh frozen plasma transfusion) | N.A | - | 0 | (0) | 8 | (24.2) |  |
| Respiratory system (need for ventilatory support) | 1 | (1.4) | 1 | (1.4) | 8 | (24.2) |  |
| Cardiovascular system (inability to keep adequate blood arterial pressure) ^‡^ | 0 | (0) | 0 | (0) | 9 | (27.3) |  |
| Gastro-intestinal system (need for enteral or parenteral feeding) | 2 | (3.1) | 1 | (1.4) | 30 | (90.9) |  |
| Hepatic (ASAT level > 50 IU/L) | N.A | - | 0 | (0) | 20 | (60.6) |  |
| Renal function (gestational age-specific abnormal urine output) | N.A | - | 0 | (0) | 4 | (12.1) |  |
| Acid base status (gestational age-specific abnormal base deficit) | N.A | - | 0 | (0) | 4 | (12.1) |  |
| **NOTE.** ^†^ lenticulo-thalamo striatal vasculitis, frontal or parietal hyperechogenicity on head ultrasound, or scattered white matter lesions on MRI scans ^‡^ volume expansion or vasopressor amines. N.A not assessed. | | | | | | | |
