# Supplementary material for: Neurocognitive Outcome of Children Exposed to Perinatal Mother-to-Child Chikungunya Virus Infection: The CHIMERE Cohort Study on Reunion Island
Source: PLoS Negl Trop Dis. 2014 Jul 17;8(7):e2996. doi: 10.1371/journal.pntd.0002996 (PMC4102444; doi:10.1371/journal.pntd.0002996)
Supplement: Table S4 — Predictors of global neurodevelopmental delay in bivariable Poisson regression analysis, CHIMERE cohort, Reunion island, 2008. Developmental quotients (DQ) were measured between 15.8 and 27 months of age. § Global neurodevelopmental delay (GND) is defined for DQ≤85. Data are numbers, percentages, crude IRR (incidence rate ratio) and 95% confidence intervals. ¶ P values are given for crude Wald tests. † This propensity score is derived from maternal population (see table 2 of ref. [12]) assigning positive or negative points to rounded-value beta coefficients associated with categories of maternal origin, education, marital status, parity and body mass index; ‡ gestational age <37 weeks; #<10th percentile of AUDIPOG growth charts; *corrected for 24 months postnatal age. (DOCX) [file pntd.0002996.s004.docx]

**Supporting file 4**

| **Table S4. Predictors of global neurodevelopmental delay in bivariable Poisson regression analysis, CHIMERE cohort, Reunion island, 2008** | | | | | | |
| --- | --- | --- | --- | --- | --- | --- |
| **Parental characteristics** | **Total** | **Children with GND** ^§^ | | **Crude IRR** | **(95% CI)** | ***P* value ^¶^** |
| Paternal age |  |  |  |  |  | 0.110 |
| ≤ 20 years | 6 | 0 | (0.0) | 0.54 | (0.08 - 3.41) |  |
| 21 to 30 years | 52 | 15 | (28.8) | 1 | - |  |
| 31 to 40 years | 64 | 6 | (9.4) | 0.41 | (0.18 - 0.87) |  |
| > 40 years | 19 | 6 | (31.6) | 1.03 | (0.47 - 2.24) |  |
| Maternal age |  |  |  |  |  | 0.179 |
| ≤ 20 years | 14 | 4 | (28.6) | 1.12 | (0.45 - 2.75) |  |
| 21 to 30 years | 94 | 24 | (25.5) | 1 | - |  |
| 31 to 40 years | 56 | 8 | (14.3) | 0.56 | (0.26 - 1.16) |  |
| > 40 years | 4 | 2 | (50.0) | 1.96 | (0.69 - 5.55) |  |
| 5-item social deprivation score ^†^ |  |  |  |  |  | 0.083 |
| Low (-1 to 0 point) | 35 | 4 | (11.4) | 1 | - |  |
| Moderate (1 to 2 points) | 84 | 18 | (21.4) | 1.87 | (0.68 - 5.16) |  |
| High (3 to 7 points) | 49 | 16 | (32.6) | 2.86 | (1.04 - 7.84) |  |
| **Neonatal characteristics** | **Total** | **Children with GND** ^§^ | | **Crude IRR** | **(95% CI)** | ***P* value ^¶^** |
| Chikungunya virus infection |  |  |  |  |  | < 0.001 |
| Yes | 33 | 17 | (51.5) | 3.31 | (1.97 - 5.54) |  |
| No | 135 | 21 | (15.6) | 1 | - |  |
| Preterm birth ^‡^ |  |  |  |  |  | 0.037 |
| Yes | 14 | 6 | (42.9) | 2.06 | (1.04 - 4.08) |  |
| No | 154 | 32 | (20.8) | 1 | - |  |
| Small for gestational age ^♯^ |  |  |  |  |  | 0.080 |
| Yes | 29 | 10 | (34.5) | 1.71 | (0.93 - 3.13) |  |
| No | 139 | 28 | (20.1) | 1 | - |  |
| 5-minute Apgar score |  |  |  |  |  | 0.476 |
| < 10 | 17 | 5 | (29.4) | 1.34 | (0.60 - 2.97) |  |
| 10 | 150 | 33 | (22.0) | 1 | - |  |
| Breastfeeding at discharge |  |  |  |  |  | 0.004 |
| Yes | 117 | 19 | (16.2) | 0.44 | (0.25 - 0.77) | - |
| No | 49 | 18 | (36.7) | 1 |  |  |
| Head circumference * |  |  |  |  |  | N.A |
| - 1 S.D ≤ z-score < + 2 S.D | 143 | 28 | (19.6) | 1 | - |  |
| - 2 S.D ≤ z-score < - 1 S.D | 4 | 1 | (25.0) | 1.28 | (0.22 - 7.24) |  |
| z-score < - 2 S.D | 4 | 4 | (100) | 5.11 | (3.65 - 7.13) |  |
| Head growth |  |  |  |  |  | 0.027 |
| - 1 S.D ≤ z-score < + 2 S.D | 129 | 26 | (20.2) | 1 | - |  |
| - 2 S.D ≤ z-score < - 1 S.D | 19 | 5 | (26.3) | 1.31 | (0.56 - 2.99) |  |
| z-score < - 2 S.D | 3 | 2 | (66.7) | 3.31 | (1.38 - 7.92) |  |
| **NOTE.** Developmental quotients (DQ) were measured between 15.8 and 27 months of age. ^§^ Global neurodevelopmental delay (GND) is defined for DQ ≤85.  Data are numbers, percentages, crude IRR (incidence rate ratio) and 95% confidence intervals.  ^¶^ *P* values are given for crude Wald tests.  ^†^ This propensity score is derived from maternal population (see table 2 of ref. [12]) assigning positive or negative points to rounded-value beta coefficients associated with categories of maternal origin, education, marital status, parity and body mass index; ^‡^ gestational age < 37 weeks; ^#^< 10^th^ percentile of AUDIPOG growth charts; *corrected for 24 months postnatal age. | | | | | | |
