# Supplementary material for: Neurocognitive Outcome of Children Exposed to Perinatal Mother-to-Child Chikungunya Virus Infection: The CHIMERE Cohort Study on Reunion Island
Source: PLoS Negl Trop Dis. 2014 Jul 17;8(7):e2996. doi: 10.1371/journal.pntd.0002996 (PMC4102444; doi:10.1371/journal.pntd.0002996)
Supplement: Table S5 — Predictors of global neurodevelopmental delay in three alternative multivariable regression models: GLM Log-Binomial, GEE-Logistic and Proportional hazard model, CHIMERE cohort, Reunion island, 2008. Developmental quotients (DQ) were measured between 15.8 and 27 months of age. § Global neurodevelopmental delay (GND) is defined for DQ≤85. † see ref. [16] for precisions; ‡ see ref. [17] for precision. Data are numbers, percentages, adjusted RR (risk ratios), adjusted OR (odds ratios) and adjusted HR (hazard ratios), and robust SE (robust standard error). ¶ P values are given for adjusted Wald tests. The model is adjusted for the social deprivation propensity score (see table 2 of ref. [12]) assigning positive or negative points to the rounded-value beta coefficients associated with categories of maternal origin, education, marital status, parity and body mass index; small for gestational age (defined for birth-weight <10th percentile of AUDIPOG growth charts); *head circumference is corrected for 24 months of postnatal age; **head growth is taken as time-to-event covariate on a clinical and statistical basis, the value of head circumference being dependent of the timing of the measure with different growth kinetics between groups. (DOCX) [file pntd.0002996.s005.docx]

**Supporting file 5**

| **Table S5. Predictors of global neurodevelopmental delay in three alternative multivariable regression models: GLM Log-Binomial, GEE-Logistic and Proportional hazard model, CHIMERE cohort, Reunion island, 2008** | | | | | | |
| --- | --- | --- | --- | --- | --- | --- |
| **GLM Log Binomial**  ^†^ |  |  | |  |  |  |
| **Predictors** | **Total** | **Children with GND** ^§^ | | **Adjusted RR** | **(95% CI)** | ***P* value ^¶^** |
| Chikungunya virus infection |  |  |  |  |  |  |
| Yes | 32 | 16 | (50.0) | 2.92 | (1.57 - 5.41) | 0.001 |
| No | 119 | 17 | (14.3) | 1 | - | - |
| Head circumference * |  |  |  |  |  |  |
| - 1 S.D ≤ z-score < + 2 S.D | 143 | 28 | (19.6) | 1 | - | - |
| - 2 S.D ≤ z-score < - 1 S.D | 4 | 1 | (25.0) | 1.46 | (0.81 - 2.62) | 0.201 |
| z-score < - 2 S.D ^¶^ | 4 | 4 | (100) | 2.68 | (1.59 - 4.48) | < 0.001 |
| **GEE Logistic**  ^†^ |  |  | |  |  |  |
| **Predictors** | **Total** | **Children with GND** ^§^ | | **Adjusted OR** | **(95% CI)** | ***P* value ^¶^** |
| Chikungunya virus infection |  |  |  |  |  |  |
| Yes | 32 | 16 | (50.0) | 4.27 | (3.75 - 4.86) | < 0.001 |
| No | 119 | 17 | (14.3) | 1 | - | - |
| Head circumference * |  |  |  |  |  |  |
| - 1 S.D ≤ z-score < + 2 S.D | 143 | 28 | (19.6) | 1 | - | - |
| - 2 S.D ≤ z-score < - 1 S.D | 4 | 1 | (25.0) | 0.77 | (0.03 - 16.66) | 0.735 |
| z-score < - 2 S.D | 4 | 4 | (100) | 30.12 | (1.47 – 613.19) | 0.027 |
| **Proportional hazard model** ^‡^ |  |  | |  |  |  |
| **Predictors** | **Total** | **Children with GND** ^§^ | | **Adjusted HR** | **(95% CI)** | ***P* value ^¶^** |
| Chikungunya virus infection |  |  |  |  |  |  |
| Yes | 32 | 16 | (50.0) | 2.78 | (1.18 - 6.49) | 0.019 |
| No | 119 | 17 | (14.3) | 1 | - | - |
| Head growth ** |  |  |  |  |  |  |
| - 1 S.D ≤ z-score < + 2 S.D | 129 | 26 | (20.3) | 1 | - | - |
| - 2 S.D ≤ z-score < - 1 S.D | 19 | 5 | (26.3) | 1.01 | (0.96 - 1.05) | 0.785 |
| z-score < - 2 S.D | 3 | 2 | (66.7) | 1.13 | (1.05 - 1.22) | 0.001 |
| **NOTE.** Developmental quotients (DQ) were measured between 15.8 and 27 months of age. ^§^ Global neurodevelopmental delay (GND) is defined for DQ ≤85.  ^†^ see ref. [16] for precisions; ^‡^ see ref.[17] for precision.  Data are numbers, percentages, adjusted RR (risk ratios), adjusted OR (odds ratios) and adjusted HR (hazard ratios), and robust SE (robust standard error). ^¶^ *P* values are given for adjusted Wald tests.  The model is adjusted for the social deprivation propensity score (see table 2 of ref. [12]) assigning positive or negative points to the rounded-value beta coefficients associated with categories of maternal origin, education, marital status, parity and body mass index; small for gestational age (defined for birth-weight < 10^th^ percentile of AUDIPOG growth charts); *head circumference is corrected for 24 months of postnatal age; **head growth is taken as time-to-event covariate on a clinical and statistical basis, the value of head circumference being dependent of the timing of the measure with different growth kinetics between groups. | | | | | | |
