# Supplementary material for: Neurocognitive Outcome of Children Exposed to Perinatal Mother-to-Child Chikungunya Virus Infection: The CHIMERE Cohort Study on Reunion Island
Source: PLoS Negl Trop Dis. 2014 Jul 17;8(7):e2996. doi: 10.1371/journal.pntd.0002996 (PMC4102444; doi:10.1371/journal.pntd.0002996)
Supplement: Table S6 — Etiologic fractions of the predictors of global neurodevelopmental delay and overall attributable risk fraction explained by two GEE-logistic multivariable regression models, CHIMERE cohort, Reunion island, 2008. Developmental quotients (DQ) were measured between 15.8 and 27 months of age. § Global neurodevelopmental delay (GND) is defined for DQ≤85. Data are numbers, percentages, adjusted odd ratios (OR), standard errors (SE), attributable risk percents (ARP) and attributable risk fraction (ARF). The effect of CHIKV infection on GND is confounded for adjusted OR change >20% of its crude value (adjusted OR <4.60). The amount of residual confounding is measured as the inverse of the ARF of the model: RC = 1- ARF (%). Overmatching is defined for an adjusted OR change of 10–20% of its crude value with concomitant SE change >20% (SE out of 1.95–2.92). †The models are adjusted for the social deprivation propensity score (see table 2 of ref. [12]) assigning positive or negative points to the rounded-value beta coefficients associated with categories of maternal origin, education, marital status, parity and body mass index; small for gestational age (defined for birth-weight <10th percentile of AUDIPOG growth charts); *head circumference is corrected for 24 months of postnatal age or ‡ gestational age <37 weeks. (DOCX) [file pntd.0002996.s006.docx]

**Supporting file 6**

| **Table S6. Etiologic fractions of the predictors of global neurodevelopmental delay and overall attributable risk fraction explained by two GEE-logistic multivariable regression models, CHIMERE cohort, Reunion island, 2008** | | | | | | | | | | | | |  |  |  |
| --- | --- | --- | --- | --- | --- | --- | --- | --- | --- | --- | --- | --- | --- | --- | --- |
| **Bivariate analysis** | **Total** | **Children with GND** ^§^ | | | | **Crude OR** | | **(95% CI)** | **SE** | | **Crude ARP** | | | |  |
| Chikungunya virus infection |  |  |  | | |  | |  |  | |  | | | | |
| Yes | 33 | 17 | (51.5) | | | 5.77 | | (2.51 - 13.21) | 2.44 | | 69.8 % | | | | |
| No | 135 | 17 | (15.6) | | | 1 | |  |  | |  | | | | |
| **Model A. Control for head circumference (n=151) : unnecessary adjustment and maximal residual confounding** | | | | | | | | | | | | | | | |
| **Predictors** | **Total** | **Children with GND** ^§^ | | | | **Adjusted OR** | | **(95% CI)** | | **SE** | **Adjusted ARP** | | |  |  |
| 5-item social deprivation score ^†^ |  |  |  | | |  | |  | |  |  | | |  |  |
| Low (-1 to 0 point) | 33 | 4 | (12.1) | | | 1 | |  | |  |  | | |  |  |
| Moderate (1 to 2 points) | 73 | 14 | (19.2) | | | 1.95 | | (1.47 – 2.58) | | 1.33 | 42.8 % | | |  |  |
| High (3 to 7 points) | 45 | 15 | (33.3) | | | 2.52 | | (0.92 – 6.90) | | 1.91 | 53.0 % | | |  |  |
| Chikungunya virus infection |  |  |  | | |  | |  | |  |  | | |  |  |
| Yes | 32 | 16 | (50.0) | | | 4.27 | | (3.75 - 4.86) | | 2.11 | 65.6 % | | |  |  |
| No | 119 | 17 | (14.3) | | | 1 | |  | |  |  | | |  |  |
| Small for gestational age ^♯^ |  |  |  | | |  | |  | |  |  | | |  |  |
| Yes | 27 | 9 | (33.3) | | | 1.83 | | (1.26 – 2.68) | | 0.97 | 36.5 % | | |  |  |
| No | 124 | 24 | (19.5) | | | 1 | |  | |  |  | | |  |  |
| Head circumference * |  |  |  | | |  | |  | |  |  | | |  |  |
| - 1 S.D ≤ z-score < + 2 S.D | 143 | 28 | (19.6) | | | 1 | |  | |  |  | | |  |  |
| - 2 S.D ≤ z-score < - 1 S.D | 4 | 1 | (25.0) | | | 0.77 | | (0.03 - 16.66) | | 1.61 | - 24.0 % | | |  |  |
| z-score < - 2 S.D | 4 | 4 | (100) | | | 30.12 | | (1.47 – 613.19) | | - | 77.7 % | | |  |  |
| **Pool of predictors** |  |  |  | | | |  |  | |  | **Adjusted ARF** | | |  |  |
| Four covariates |  |  |  |  | | |  |  | |  | 94.9 % | | |  |  |
| **Model B. Control for preterm birth (n=168) : absence of overadjustment and minimal residual confounding** | | | | | | | | | | | | |  |  |  |
| **Predictors** | **Total** | **Children with GND** ^§^ | | | **Adjusted OR** | | | **(95% CI)** | | **SE** | **Adjusted ARP** | | |  |  |
| 5-ite m social deprivation score ^†^ |  |  |  | |  | | |  | |  |  | | |  |  |
| Low (-1 to 0 point) | 35 | 4 | (11.4) | | 1 | | |  | |  |  |  | |  |  |
| Moderate (1 to 2 points) | 84 | 18 | (21.4) | | 2.00 | | | (0.68 – 5.80) | | 1.16 | 44.3 % | | |  |  |
| High (3 to 7 points) | 49 | 16 | (32.6) | | 2.34 | | | (1.09 – 4.98) | | 1.47 | 50.7 % | | |  |  |
| Chikungunya virus infection |  |  |  | |  | | |  | |  |  | | |  |  |
| Yes | 33 | 17 | (51.5) | | 5.20 | | | (5.07 - 5.33) | | 2.36 | 68.2 % | | |  |  |
| No | 135 | 17 | (15.6) | | 1 | | |  | |  |  | | |  |  |
| Preterm birth ^‡^ |  |  |  | |  | | |  | |  |  | | |  |  |
| Yes | 14 | 6 | (42.9) | | 2.93 | | | (2.85 – 3.00) | | 1.94 | 52.1% | | |  |  |
| No | 154 | 32 | (20.8) | | 1 | | |  | |  |  | | |  |  |
| Small for gestational age ^♯^ |  |  |  | |  | | |  | |  |  | | |  |  |
| Yes | 29 | 9 | (34.5) | | 2.16 | | | (1.52 -3.07) | |  | 42.9 % | | |  |  |
| No | 139 | 28 | (20.1) | | 1 | | |  | | 1.06 |  | | |  |  |
| **Pool of predictors** |  |  |  | |  | | |  | |  | **Adjusted ARF** | | |  |  |
| Four covariates |  |  |  | |  | | |  | |  | 97.7 % | | |  |  |
| **NOTE.** Developmental quotients (DQ) were measured between 15.8 and 27 months of age. ^§^ Global neurodevelopmental delay (GND) is defined for DQ ≤85.  Data are numbers, percentages, adjusted odd ratios (OR), standard errors (SE), attributable risk percents (ARP) and attributable risk fraction (ARF).  The effect of CHIKV infection on GND is confounded for adjusted OR change > 20% of its crude value (adjusted OR < 4.60). The amount of residual confounding is measured as the inverse of the ARF of the model: RC = 1- ARF (%). Overmatching is defined for an adjusted OR change of 10-20% of its crude value with concomitant SE change > 20% (SE out of 1.95-2.92).  ^†^The models are adjusted for the social deprivation propensity score (see table 2 of ref. [12]) assigning positive or negative points to the rounded-value beta coefficients associated with categories of maternal origin, education, marital status, parity and body mass index; small for gestational age (defined for birth-weight < 10^th^ percentile of AUDIPOG growth charts); *head circumference is corrected for 24 months of postnatal age or ^‡^ gestational age < 37 weeks. | | | | | | | | | | | | |  |  |  |
